# Estimating the cost of referral and willingness to pay for referral to higher-level health facilities: a case series study from an integrated community case management programme in Uganda

**DOI:** 10.1186/s12913-015-1019-5

**Published:** 2015-08-28

**Authors:** Agnes Nanyonjo, Benson Bagorogoza, Frida Kasteng, Godfrey Ayebale, Fredrick Makumbi, Göran Tomson, Karin Källander, for the inSCALE study group

**Affiliations:** Department of Public Health Sciences, Karolinska Institutet, Tomtebodavägen 18 A, SE-171 77 Stockholm, Sweden; Malaria Consortium Uganda Office, P.O Box 8045, Kampala, Uganda; Department of Global Health and Development, London School of Hygiene and Tropical Medicine, Keppel Street, WC1E 7HT London, United Kingdom; Department of Epidemiology and Biostatistics, Makerere University School of Public Health, P.O Box 7062, Kampala, Uganda; Malaria Consortium, Development House, 56-64 Leonard Street, London, EC2A 4LT UK; Medical Management Centre (MMC), Department of Public Health Sciences, Karolinska Institutet, Tomtebodavägen 18 A, 17177 Stockholm, Sweden

## Abstract

**Background:**

Integrated community case management (iCCM) relies on community health workers (CHWs) managing children with malaria, pneumonia, diarrhoea, and referring children when management is not possible. This study sought to establish the cost per sick child referred to seek care from a higher-level health facility by a CHW and to estimate caregivers’ willingness to pay (WTP) for referral.

**Methods:**

Caregivers of 203 randomly selected children referred to higher-level health facilities by CHWs were interviewed in four Midwestern Uganda districts. Questionnaires and document reviews were used to capture direct, indirect and opportunity costs incurred by caregivers, CHWs and health facilities managing referred children. WTP for referral was assessed through the ‘bidding game’ approach followed by an open-ended question on maximum WTP. Descriptive analysis was conducted for factors associated with referral completion and WTP using logistic and linear regression methods, respectively. The cost per case referred to higher-level health facilities was computed from a societal perspective.

**Results:**

Reasons for referral included having fever with a negative malaria test (46.8 %), danger signs (29.6 %) and drug shortage (37.4 %). Among the referred, less than half completed referral (45.8 %). Referral completion was 2.8 times higher among children with danger signs (*p* = 0.004) relative to those without danger signs, and 0.27 times lower among children who received pre-referral treatment (*p* < 0.001). The average cost per case referred was US$ 4.89 and US$7.35 per case completing referral. For each unit cost per case referred, caregiver out of pocket expenditure contributed 33.7 %, caregivers’ and CHWs’ opportunity costs contributed 29.2 % and 5.1 % respectively and health facility costs contributed 39.6 %.

The mean (SD) out of pocket expenditure was US$1.65 (3.25). The mean WTP for referral was US$8.25 (14.70) and was positively associated with having received pre-referral treatment, completing referral and increasing caregiver education level.

**Conclusion:**

The mean WTP for referral was higher than the average out of pocket expenditure. This, along with suboptimal referral completion, points to barriers in access to higher-level facilities as the primary cause of low referral. Community mobilisation for uptake of referral is necessary if the policy of referring children to the nearest health facility is to be effective.

## Background

Malaria, pneumonia and diarrhoea are among the leading killer diseases among children aged five years and below. Efforts in realizing the Millennium Development Goals are tailored towards tackling the three diseases at community level through integrated community case management (iCCM) [1–3]. iCCM involves diagnosis and treatment of malaria, pneumonia and diarrhoea by community health workers (CHWs) in addition to referral of severely sick children, children whose conditions cannot be treated by CHWs, and newborns. However, slow implementation of the approach has been noted in the 75 ‘countdown’ countries which together account for more than 95 % of all maternal, newborn, and childhood mortality [3].

One key component of integrated community approaches is a well functional referral system which has the ability to send referred cases to higher-level facilities [4]. However, not all children referred to obtain care from higher-level facilities access referral treatment, indicating a gap in health services access [5–7]. Previous studies have highlighted the challenges faced by caregivers of children referred, including problematic transport options, lack of money, competing responsibilities, perceived poor quality of care at the health facility and improvement in the child’s condition following pre-referral treatment [6–8]. There is general agreement that the cost of referral must decrease to make referral completion an achievable goal [7]. Moreover, the limited ability of first level health facilities to handle severely ill children in sub-Saharan Africa has been documented [9, 10]. There is also a general concern about the high out of pocket expenditure for health care among households in resource-poor settings, particularly health care sought from higher-level facilities [11, 12]. Furthermore, people are often willing to pay (WTP) for western medicine if the cause of disease is perceived to be biomedical [13]. However WTP does not translate into actual expenditure; as the ability to pay is influenced by other factors. One of the factors that further limit low-income earner’s ability to pay for biomedical treatment, despite WTP, is the lack of flexibility in how and when fees should be paid, as more flexible payment options would allow time to save or accrue the funds needed [13]. The Uganda iCCM guidelines dictate that children with danger signs seen by a CHW should be referred to the nearest heath centre following pre-referral treatment for suspected severe malaria, pneumonia, dehydration or other danger signs in young children and newborns. Caregivers of children are likely to incur recurrent costs if the first referral centre is not able to handle complicated cases. The mean out of pocket expenditure for a completed referral was estimated to be US$11 in a study conducted in Uganda in 2003, a value that is way too costly for an average caregiver from rural Uganda (7).

The evaluation of health care programmes and of the economic burden of disease is most meaningful when examined from a societal viewpoint, considering all costs, regardless of to whom they accrue, i.e. both health system and household costs, however studies with such analysis design are still scanty in low-income countries [14, 15]. The contingent valuation method, which elicits people’s WTP, can be used to measure the value attached to a health service delivery process, even in low-income countries [16–18]. The stated WTP for referral can therefore estimate a caregivers’ valuation of referral and can be used as a basis for financial incentives fostering referral completion. This study used a societal perspective to examine average cost per child referred to a higher-level health facility as well as WTP for referral among the caregivers of the referred children. Such an approach is useful in identifying the brunt of costs borne by child caregivers in relation to service providers and can be used for planning interventions for a functional and integrated community referral system devoid of financial and economic barriers to referral completion.

## Methods

### Setting

The study was conducted in early 2013 in four of the nine mid-western districts of Uganda where iCCM had been implemented since 2010. Based on records from the Uganda health facility inventory [19], the area was registered to have a total of 276 health centres; 192 government owned, 51 private not for profit and 33 private health centres (HCs), serving a catchment area population of 2.2 million people (18 % aged < 5 years). The nomenclature of the health services in Uganda is based on the services they provide and the catchment area they are intended to serve. In Uganda’s health system hierarchy, the lowest level health centre operating at the village level (HC I) is the CHW who works from home [19]. Among the HCs with a physical structure, the lowest level is a HC II located at the parish level that offers outpatient services through a comprehensive nurse, followed by HC III offering in-patient services through clinical officers at the sub-county level. HC IVs located at the county level have doctors who offer in-patient services while hospitals located at the district level offer specialist services. All health centres offer basic health promotive and curative services and staff from higher level HCs supervise their peers at the lower level HCs. Patients with complex disease conditions are referred to either the regional or national referral hospitals offering more comprehensive specialist services [19]. However, alternative health service providers such as private clinics, drug shops and traditional healers, all without designated catchment areas are common in the study area. On average most of the HCs with a physical structure in the study area were level II or III and most villages had at least two CHW trained on delivering iCCM.

According to Uganda’s village health team (VHT) strategy, two CHWs (locally referred to as VHT members) in each village should be trained on iCCM in addition to regular health education and promotion activities. They are provided with job aids and are entrusted with the role of diagnosing and treating children between 2–59 months with uncomplicated disease whilst referring those with danger signs, such as convulsions, unconsciousness, chest in-drawing, excessive vomiting and chronic conditions. Additionally, CHWs are expected to offer safe referrals for children they cannot treat, such as sick newborns and children who do not fall within 2–59 months age bracket. In the study area, CHWs diagnosed malaria and ‘fast breathing’ pneumonia with the help of a simple rapid diagnostic test and respiratory timer respectively. They treated malaria with artementher-lumefantrine combination therapy, pneumonia with amoxicillin and diarrhoea with a combination of ORS and zinc. They were also required to offer pre-referral rectal artesunate to children with signs of severe malaria, artementher-lumefantrine to children with fevers lasting more than 7 days, ORS to children with diarrhoea, and amoxicillin to children with signs of severe pneumonia before referral to the nearest health facility.

### Study design and procedures

**Fig. 1:**
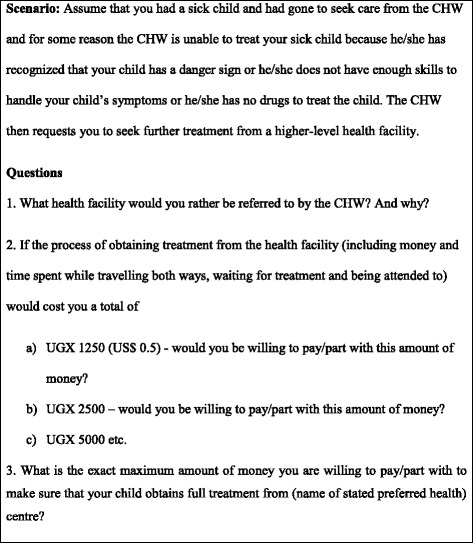
Bidding game scenario

Data were drawn from a sample of caregivers of sick children referred to seek care from higher- level healthcare facilities by CHWs. The sample size was limited to children who had been referred within two weeks of the interview. The desired sample size was calculated in relation to the expected mean cost of referral. However, at the time of the study, there were no studies on referral from which mean societal costs could be estimated. There were two existing studies conducted in Uganda addressing higher-level facility referrals which provided only median estimates of out of pocket expenditures incurred by child caregivers [7, 8]. In the absence of suitable estimates for societal costs of referral, the sample size was estimated using the best alternative available evidence. Therefore, the sample size was estimated using the mean out of pocket expenditure as a proxy for total costs. The mean out of pocket expenditure was calculated from the dataset of the Kallander et al. 2006 study [8]. Using the sampsi command in STATA 12 (StataCorp LP, College Station, TX), assuming a mean of 8950 UGX, a standard deviation of 16,860 UGX [8], a hypothesised mean referral cost of 15,000 UGX in the sample (given current inflation and cost of living), and adjusting for cluster design effects and a loss to follow up of 5 %, a total of 174 referred children was required to detect the mean cost per completed referral equivalent to the hypothesised mean. With the village as the primary sampling unit and assuming uniform distribution, four districts were selected out of nine districts. Twelve sub-counties were selected from a list of all the sub-counties in the four selected districts. Under the assumption that a CHW would have referred at least one child within two weeks of interview, 50 villages were sampled from the list of sub-counties using probability proportional to population size of the sub-county. Referred children were identified through an interview with CHWs from the selected villages. Caregivers of referred children were traced with the help of the CHW and were given a facilitator-administered questionnaire by a trained research assistant. Most parts of the questionnaire were adapted from previous research on referral in integrated management of childhood illnesses and home based management of fever [7–9]. One part of the questionnaire assessed socio-demographic characteristics of the referred child and his/her caregiver. Another part of the questionnaire assessed the referral process with special emphasis on the circumstances under which the child was referred, and the steps taken by the caregiver thereafter. Referral completion was operationally defined as receiving care from a recognised iCCM referral health centre supervising the CHWs. Caregivers obtaining care from non-iCCM designated health facilities were classified as incomplete referrals warranting further investigation. The questionnaire had a set of open-ended questions aimed at establishing both direct costs, such as monetary expenses incurred by the caregiver during the referral processes, as well as non-financial costs, such time spent on caring for the sick child. In order to establish the importance caregivers attached to following referral advice from CHWs to seek health care from higher-level facilities, contingent valuation methods were used to elicit caregivers WTP for referral [16]. Caregivers were asked about their preferred referral health facilities and the reasons as to why they preferred these health facilities. The ‘bidding game’ followed by an open-ended question on maximum WTP was chosen as the method of elicitation. This was because the culture of bargaining for services (including health services) is popular in Uganda making it easy for participants to understand the ‘bidding game’ [18]. Additionally, the method has been shown to be reliable in eliciting WTP [20]. While assessing the WTP for referral using the ‘bidding game’, participants were presented with a scenario where they were supposed to assume they had a sick child and had gone to seek care from the CHW. However, on this particular occasion the CHW was unable to treat their sick child because he or she had noticed that the child had a danger sign, or he or she did not have enough skills to handle the child’s symptoms, or he/she had no drugs to treat the child. The CHW then requests the caregiver to seek further treatment from his or her own previously stated preferred health facility. The respondent was then asked if he/she would take his/her child to the preferred referral health facility if it would cost him/her a total of US$0.5 in costs related to the process of seeking and obtaining full treatment. The starting bidding value of US$0.5 was estimated from interviews with a small sample of participants. If the participant answered yes to the bid, the price was raised to US$1.0 and then US$2.0 etc. on condition that the participant accepted the previous level of WTP. On rejecting a higher bid, the respondent was asked to mention the maximum amount that he/she would be willing to pay for referral (Fig. 1). This was done in order to give the respondents an opportunity to give a more specific value of WTP.

The study adopted a societal viewpoint [16] while estimating the cost of referral. Therefore, a costing matrix capturing all costs (financial and economic) related to management of children referred, regardless of to whom they accrued, was designed. CHW associated costs were captured through a questionnaire that assessed time spent managing children who required referral and resources utilised. The opportunity costs to the volunteering CHWs and the child caregivers for this time were estimated through market price value approaches [16]. In these approaches, the time of volunteers is valued by a relevant market wage rate. Since Uganda is a low-income country that does not have a perfect competitive labour market due to high levels of unemployment, it was important to make assumptions about CHW and caregiver earnings. In the presence of an imperfect labour market, the literature often recommends choosing among minimum wage approaches, average labourer wage and time foregone as reported by the participants [16, 21, 22]. The value of time spent by CHWs and caregivers while working with and caring for referred children, respectively, was therefore estimated in terms of foregone monthly income using an average income for rural dwellers as reported in the Uganda National household survey [23]. This is because there is no minimum wage policy in Uganda and the average income foregone could not be estimated by some of the participants in this study due to limitations in the questionnaire. There were limited health facility costing studies conducted in Uganda, the best available evidence was from a study by Medical Sciences for Health (MSH) in which the minimum health care package at health centres in Uganda was costed [24]. The MSH study used the cost and revenue plus (CORE Plus) analysis tool to model direct and indirect costs associated with delivery of a health services by service protocol. Indirect costs were defined as costs that cannot be traced to one particular service. The researchers computed the cost per service delivered by taking the standard unit cost (staff, drugs and medical supplies and lab tests), and loading the indirect and operating costs proportionally across the services. The MSH study however excluded capital costs and depreciation costs since they are not normally part of the recurrent budgets of the health facilities. Therefore, direct and indirect health facility related costs were identified using the estimates available from the MSH study, Table 1 shows a summary of the exhaustive costs included in the matrix as well as the data sources.

**Table 1:** Summary of type and source of costs explored per level

	Type of cost	Caregiver level	CHW level	Health facility level
1	Treatment costs/service delivery related direct and indirect costs	User fees, drug costs, costs of diagnostic tests and other medical supplies, Cost of hospitalisation	Drug cost	Drugs, diagnostics and medical supply costs (outpatient or inpatient management costs)
			Cost of diagnostics and other supplies	
2	Transport costs	Transportation money	Cost of facilitated referral	Cost of transporting patient to higher level facility
	Data source	Caregiver questionnaire	CHW questionnaire	Literature
3	Journey related costs	Any other costs incurred due to the journey e.g. accommodation and food	N/A	N/A
	Data source	Caregiver questionnaire	N/A	N/A
4	Time costs	Opportunity cost of taking a child referral centre, waiting time and consultation time, hospitalisation time	Opportunity cost of time managing patient	Not captured
	Data source	Caregiver questionnaire, literature	CHW questionnaire, literature	N/A

**Table 2:** Reasons for referral and reasons for not completing referral

^a^Reason for referral	Proportion (*N* = 197)
Fever (−ve RDT)	46.8 %
No drugs	37.4 %
Danger sign	29.6 %
Other	4.9 %
Main reason for not completing referral	Proportion (*N* = 107)
Child improved	28.4 %
Facility closed	24.8 %
Long distance	21.1 %
Anticipated drug shortage	12.0 %
Facility not trustworthy	7.3 %
^b^Other	6.4 %

^a^Multiple reasons for referral possible

^b^Other mainly consisted of flu, eye conditions, and stomachache and skin rash

**Table 3:** Average cost per referred case overall and by health facility level

Type of cost/type of health facility	Overall mean (SD)	HC II mean (SD)	HC III mean (SD)	HCIV/HOSPITAL mean (SD)	% Contribution to unit cost
Per capita cost to health facility^a^	1.94	1.68	1.15	3.01	39.6
Opportunity cost CHW	0.25 (0.12)	0.24 (0.11)	0.29 (0.14)	0.19(0.08)	5.1
Opportunity cost caregiver	1.43 (2.02)	1.39 (1.91)	1.47 (1.78)	1.69 (3.10)	29.2
Out of pocket expenditure caregiver	1.65 (3.25)	1.17 (2.53)	2.04 (3.89)	1.92 (3.08)	33.7
Total unit cost	4.89	4.34	4.80	6.68	
Costs among completed referrals					
Cost borne by health facility^a^	1.94	1.68	1.15	3.01	26.3
Opportunity cost CHW	0.25 (0.12)	0.24 (0.11)	0.29 (0.14)	0.19(0.08)	3.4
Opportunity cost caregiver	2.73 (2.17)	2.62 (2.09)	2.99 (1.51)	3.81 (3.73)	37.1
Out of pocket expenditure caregiver	2.52 (3.50)	1.33 (1.95)	3.12 (4.3)	1.71 (2.38)	34.5
Total unit cost	7.35	5.89	7.63	11.32	

^a^Average obtained from literature with no standard deviation, the costs included staff, drugs and medical supplies, lab tests and indirect costs

### Statistical analysis

The overall objectives of the analysis were to estimate the cost of referral from a societal perspective, and to estimate the median caregiver WTP for referral. Direct and indirect costs associated with referral completion, were inputted into spreadsheets to estimate the average health facility type specific cost per child referred. Direct costs incurred by caregivers and CHW during referral were analysed for distribution. Non-financial costs incurred by both caregivers and volunteering CHWs in form of opportunity costs were computed basing on assumptions described below. According to the study dataset, the average stated monthly income for the caregivers who were predominantly farmers (72 %) was US$129. Most CHWs were also farmers (86 %) with an average monthly income of US$ 95.45. However the average monthly household income for rural areas (US$125.65) from the last Uganda National Household Income Survey [23] was used for calculating opportunity costs as this was deemed a more representative value. People in rural areas were assumed to work for six days a week for approximately 8 h a day. WTP values were logarithmically transformed and a linear regression model was used to identify the socio-demographic and economic factors associated with stated WTP for referral while adjusting for clustering effects at the village level. Logistic regression was used to identify which children were more likely to complete referral. Socioeconomic status was constructed through principle component analysis of ownership of household items, household construction materials, sanitation infrastructure and means of transportation; variables which are recommended by Uganda National Bureau of Statistics. The socioeconomic status variables that weighted heaviest in the analysis were house construction materials and type of toilet facility. All analyses were done in Microsoft excel and STATA version 12.

### Ethical statement

Written informed consent was obtained from child caregivers and CHWs before study participation. Institutional consent was obtained from Makerere University School of Public Health Institutional review Board and the Uganda National Council of Science and Technology (HS958).

## Results

**Fig. 2:**
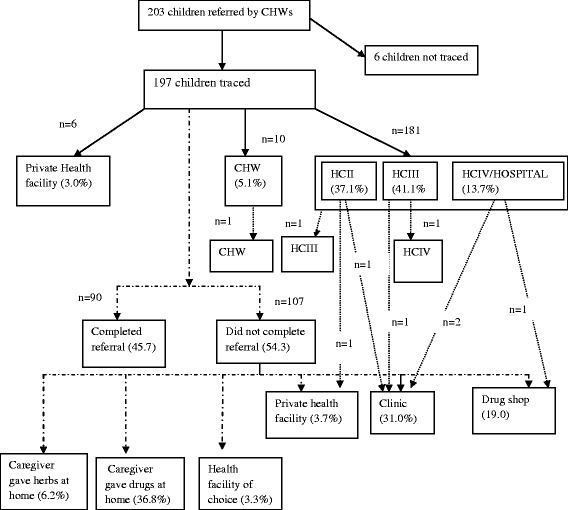
A flow diagram of the referral pathway for children referred to higher-level health facilities by community health workers (*N* = 203).  Place of first referral.  Place of second referral.  Referral completion and pathway for non compliers

Data were drawn from a sample of 203 children referred by CHWs to seek further care from the nearest health facility. The reasons for referral included danger signs, drug shortage and complaints of fever in children with a negative rapid diagnostic test for malaria (Table 2). Of the 197 traceable children, about half (54.2 %) did not complete referral. Overall, referral completion was significantly higher among children with danger signs relative to those without such signs (adjusted OR = 2.8; 95 % CI 1.4-5.4). Referral completion was also significantly lower among children who received pre-referral treatment compared to those who did not (adjusted OR = 0.27; 95 % CI 0.1-0.5). On the whole, 5.1 % of children were referred to other CHWs, 3.0 % were referred to the private sector and 91.8 % were referred to the public sector. Figure 2 provides a breakdown of the proportion of children referred to the various places and the pathways they took following referral.

Of the 181 children referred to public referral facilities 73 (40.3 %) were referred to a HC II, 81 (44.7 %) were referred to a HC III and 27 (14.9 %) were referred to a HC IV or hospital. Of the children referred to these higher-level facilities, 8 (4.45 %) were referred onwards to another health care facility. From a societal perspective, the average cost per child referred was $4.89 per child referred and USD $7.35 per child completing referral. The average cost per case referred increased with the level of health facility; from US$4.34 to US$6.68 for HC II and HC IV, respectively. Similarly the average cost per case completing referral increased from US$5.89 for HC IIs to US$11.32 for HC IVs (Table 3).

For each unit cost per case referred, caregiver out of pocket expenditure contributed 33.7 %, caregivers’ and CHWs’ opportunity costs contributed 29.2 % and 5.1 % respectively and health facility costs contributed (staff, drugs, medical supplies, lab tests and indirect costs) 39.6 %. Among the children who completed referral, caregiver out of pocket expenditure contributed 34.5 % to the unit cost per referral completed, caregivers’ and CHWs’ opportunity costs contributed 37.1 % and 3.4 % respectively and health facility costs contributed 26.3 % (Table 3). The overall median out of pocket expenditure was US$ 0 (mean US$1.65, range US$0 to US$12.45). The median out of pocket expenditure for caregivers whose children completed referral was US$1.29 (mean USD$2.52, range US$ 0 to 11.68) compared to US$0 (mean 0.92, range US$0 to US$14.56) for those who did not complete referral. Out of pocket expenditure arose from sustenance (46.3 %), transport (28.3 %) and medical fees (10.0 %). Median opportunity costs for caregivers amounted to US$0.61 (mean US$1.43, range US$ 0 to US$13.2). They varied from US$0 (mean US$0.33, range US$0 to US$19.62) for children who did not complete referral to US$2.44 (mean US$2.7, range US$0.05-US$13.12) for children who completed referral.

Among the 107 caregivers who did not complete referral for their sick children (Table 2), stated referral reasons were no drugs available (37.3 %), fever with a negative malaria test (49.1 %) and danger-signs (21.8 %). Of these, 28 % stated that the child had improved at home, 25 % reported that the health facility was closed, especially in the evenings or on the weekend, 21 % were hindered by the long distances to the health facility, 12 % anticipated a drug shortage at the health facility and 7 % felt that the staff at the health facility could not be trusted.

The mean (SD) and median (range) WTP for referral among caregivers whose children had been referred by a CHW to higher-level facilities were $8.25 (14.70) and $3.92 (0.39-157.17) respectively. Caregivers of children who completed referral had a mean WTP of $9.56 (17.20), median $5.89 (0.39-157.17) compared to a mean of $7.13 (12.13) and median $3.92 (0.39-117.87) among those who did not complete referral. The cost was positively associated with provision of pre-referral treatment, referral completion and education level of the caregiver (Table 4). Preferred referral sites for stated WTP included public facilities (52 %), private health facilities (45 %) and mission hospitals (3 %). Private facilities were preferred mainly because of having good customer care while public facilities were preferred for having inpatient services and various specialist services.

**Table 4:** Adjusted predictors of willingness to pay for referral by caregivers

Predictor	Coefficient of log WTP (95 % CI)	*p*-value
Pre-referral treatment	0.512 (0.214,0.809)	0.001*
Age of caregiver	−0.001 (−0.017,0.014)	0.820
Danger signs	−0.136 (−0.448,0.176)	0.391
Socioeconomic quintile	−0.133 (−0.331,0.065)	0.187
Education of caregiver	0.192 (0.262,0.359)	0.024*
Marital status	0.078 (−0.044,0.201)	0.209
Referral completion	0.342 (0.045,0.639)	0.024*
Sex of caregiver	−0.074 (−0.464,0.316)	0.707

*Significant at 5 % level of significance

## Discussion

The integrated community case management (iCCM) strategy relies on referral as a backup for a continuum of care for children who cannot be treated by CHWs. However, less than half of the children referred completed referral in this study. Low referral completion among febrile children with negative rapid diagnostic test has previously been reported in a study from Sierra Leone [25]. This study population is similar to that of Sierra Leone in that the fever with a negative rapid diagnostic test for malaria was the predominant cause of referral. A majority of caregivers whose children did not complete referral resorted to giving treatment at home, or went to drug shops and private clinics. This underscores the failure of the current referral policy that relies on referring children to the nearest public health facility when circumstances do not permit community case management.

Among the referred children, those who received pre-referral treatment were less likely to complete referral, compared to children who did not receive pre-referral treatment. This is consistent with a study from Tanzania [6] that discussed the dilemmas faced by caregivers following temporary improvement in a child’s condition at home. This indicates the need to establish clear referral procedures, including household follow up of children referred by CHWs, which is important in establishing strong referral systems [26]. It is also necessary to ensure that referral messages are communicated effectively by CHWs. Although iCCM stipulates that referral is between CHWs and higher-level health facilities, in a few instances there was CHW to CHW referral, CHW to private clinic referral, and health facility to private clinic referral. This should be scrutinised in the light of potential delays in access to appropriate care that might arise. CHW to CHW referral should only be encouraged if availability of drugs can be verified to the caregiver prior to referral. As much as the role of the private sector in health system strengthening cannot be underestimated [27, 28], caution should be taken as the mismanagement of patients by drug shops and clinics is widely described in the literature [29].

WTP methods have been used in low-income country context to establish demand for health services as well as to justify subsidies on commodities [18, 30, 31]. Median WTP for referral was higher than average out of pocket expenditure. Median WTP for referral was also higher than average out of pocket expenditure combined with opportunity costs incurred by caregivers. WTP was positively associated with provision of pre-referral treatment, referral completion and education level of the caregiver. The presence of a high median WTP for referral compared to both out of pocket expenditure and opportunity costs combined implies that it is not caregiver attitude towards that is primarily affecting referral completion, but health system factors such as long distances to health facilities. This is backed up by the finding that caregivers of children who received pre-referral treatment were likely to have higher WTP for referral even though they were less likely to complete referral.

Overall total out of pocket expenditure constituted a significant proportion of the total cost among children referred to higher-level health facilities. Previous studies on community referral and referral between health facilities in African contexts have expressed the need to lower the cost of referral [7, 8] and to improve quality of care at referral facilities [9, 32–34] in order to make referral an achievable goal. Although the overall out of pocket expenditures incurred by caregivers in this study were generally lower than those in previous studies [8] child caregivers still absorbed a considerable amount of the referral cost. The lower out of pocket expenditure might be an indication of improved financial and geographical access in iCCM areas since CHWs counsel caregivers to take children to the nearest health facility. The cost per child completing referral increased with the level of health facility. However this variability in cost by health centre level needs to further be scrutinised as they may indicate variability in quality of care provided at the various levels of health facilities [24].

Study limitations include the dearth of studies presenting the societal costs of referral from which the sample size could be estimated however the best available proxy (out of pocket expenditure) was used to calculate the sample size. There was a possibility of recall bias, which we tried to minimise by using a two weeks recall period. Another limitation is that the study relied on the average national income for rural dwellers to calculate assumptions about earnings of CHWs. However this is justifiable as other methods such as reported income foregone can prove difficult to validate in rural settings [22]. The health facility costs were extrapolated from a study that excluded capital costs and depreciation costs. We also made assumptions about the time worked by CHWs based on the official working time of a typical Ugandan, which may not be true for all CHWs. The ‘bidding game’ has been criticised for introducing a starting point bias and the open-ended technique has been questioned, as patients who are naive about a health care programme may not be able to attach a valid value to it [35, 36]. The starting price in this study was established through interviews with a small sample of participants. It was subsequently made as low as possible, was the same for all participants and was followed by an open-ended question on maximum WTP. The cost that a person claims to be willing to pay may deviate from what they would actually be willing to pay should they be confronted with the actual situation. However the WTP interview was conducted among only referred children who were given a realistic choice of their preferred health centre of referral.

## Conclusion

There was a suboptimal referral completion rate among children referred by CHWs to higher-level facilities. While caregivers’ out of pocket expenditure contributes significantly to the cost of referral, the mean WTP for referral was higher than the average out of pocket expenditure spent by most caregivers. This suggests that if appropriate continuum of care depends on referral to the nearest health facility as the sole support plan for children who cannot be managed by CHWs, then strategies are needed that can increase compliance with referral. Removal of other access barriers, such as transport costs and medical fees, may be necessary.

## Abbreviations

iCCM: Integrated community case management
CHWs: Community health workers
WTP: Willingness to pay
VHT: Village health team
CORE: Plus- costs and revenue
MSH: Medical Sciences for Health
